# An Analysis of the Potential Impact of Climate Change on Dengue Transmission in the Southeastern United States

**DOI:** 10.1289/EHP218

**Published:** 2016-10-07

**Authors:** Melinda K. Butterworth, Cory W. Morin, Andrew C. Comrie

**Affiliations:** 1 Department of Environmental and Earth Sciences, Willamette University, Salem, Oregon, USA; 2 Department of Global Health, University of Washington, Seattle, Washington, USA; 3 School of Geography and Development, University of Arizona, Tucson, Arizona, USA

## Abstract

**Background:**

Dengue fever, caused by a mosquito-transmitted virus, is an increasing health concern in the Americas. Meteorological variables such as temperature and precipitation can affect disease distribution and abundance through biophysical impacts on the vector and on the virus. Such tightly coupled links may facilitate further spread of dengue fever under a changing climate. In the southeastern United States, the dengue vector is widely established and exists on the current fringe of dengue transmission.

**Objectives:**

We assessed projected climate change–driven shifts in dengue transmission risk in this region.

**Methods:**

We used a dynamic mosquito population and virus transmission model driven by meteorological data to simulate *Aedes aegypti* populations and dengue cases in 23 locations in the southeastern United States under current climate conditions and future climate projections. We compared estimates for each location with simulations based on observed data from San Juan, Puerto Rico, where dengue is endemic.

**Results:**

Our simulations based on current climate data suggest that dengue transmission at levels similar to those in San Juan is possible at several U.S. locations during the summer months, particularly in southern Florida and Texas. Simulations that include climate change projections suggest that conditions may become suitable for virus transmission in a larger number of locations and for a longer period of time during each year. However, in contrast with San Juan, U.S. locations would not sustain year-round dengue transmission according to our model.

**Conclusions:**

Our findings suggest that Dengue virus (DENV) transmission is limited by low winter temperatures in the mainland United States, which are likely to prevent its permanent establishment. Although future climate conditions may increase the length of the mosquito season in many locations, projected increases in dengue transmission are limited to the southernmost locations.

**Citation:**

Butterworth MK, Morin CW, Comrie AC. 2017. An analysis of the potential impact of climate change on dengue transmission in the southeastern United States. Environ Health Perspect 125:579–585; http://dx.doi.org/10.1289/EHP218

## Introduction

The impacts of climate and climate change on infectious disease dynamics, distribution, and spread have been the subject of significant discussion (Brisbois and Ali 2010; Chaves and Koenraadt 2010; Lafferty 2009; Ostfeld 2009; Pascual and Bouma 2009; Randolph 2009) because temperature can have an impact on the seasonality and intensity of infectious disease transmission (Harvell et al. 2002). Dengue fever, caused by a virus spread predominantly by *Aedes aegypti* mosquitoes, is emblematic of a disease whose rapid expansion may be partly fueled by changing climatic conditions. Bhatt et al. (2013) estimated that the four dengue serotypes (DEN-1, 2, 3, 4) cause 390 million annual infections, 96 million of which are symptomatic, representing a significant global disease burden. Therefore, understanding links between local and global climate and weather patterns and disease outbreaks is an important topic of inquiry.

Dengue virus (DENV) transmission predominantly occurs in tropical regions. However, there has been an increase in the outbreak intensity and spatial distribution of dengue in the Americas over the past decade (Bouri et al. 2012; Brathwaite Dick et al. 2012). Travel within and across countries, the growth of substandard urban conditions, and the cessation of public health and vector control programs are collectively understood as contributing to this increase (Gubler and Clark 1995; Wilder-Smith and Gubler 2008). The possibility of concurrent circulation of up to four DEN serotypes in a given location (Halstead 1992) and changing environmental conditions further complicates dengue ecology. Laboratory and field-based studies indicate that climate has an important role in mosquito and dengue viral development and transmission; however, linking these variables to actual outbreaks remains challenging.

Meteorological variables such as temperature and precipitation affect the biophysical functioning of the mosquito and the breeding habitat (e.g., Christophers 1960). Precipitation can increase vector density by providing breeding habitat (Moore et al. 1978), and temperature affects mosquito hatching rate, development time (Mohammed and Chadee 2011), and survival (Tun-Lin et al. 2000). Temperature further influences virus transmission dynamics by shortening the extrinsic incubation period (EIP). Watts et al. (1987) reported that the EIP for the DEN-2 serotype declines from 12 days at temperatures ≤ 30°C to 7 days at 32–35°C. Similarly, Rohani et al. (2009) noted that mosquitoes became infectious with DEN-2 and DEN-4 at day 5 at 30°C, 4 days sooner than mosquitoes held at 26°C and 28°C. A shorter EIP heightens the transmission potential to humans and the outbreak intensity (Jetten and Focks 1997; Patz et al. 1998). These biophysical processes underpin the links between climate variability and observed dengue fever cases across multiple spatiotemporal scales (Cazelles et al. 2005; Gagnon et al. 2001; Hopp and Foley 2003; Johansson et al. 2009) and have led to concerns that virus transmission may increase in regions where it has until now been uncommon (Patz et al. 1998). Recent empirical work in Mexico found a decreasing number of *Ae. aegypti* with increased elevation along an altitudinal transect (Lozano-Fuentes et al. 2012). That study also found populations of *Ae. aegypti* at higher elevations in Mexico than previously recorded within the country. Collectively, the results suggest the possibility of future temperature-induced *Ae. aegypti* expansion into areas beyond current geographical boundaries.

Several model-based studies have focused on relationships between climate and dengue, highlighting the utility of these approaches (Bannister-Tyrrell et al. 2013; Focks et al. 1993; Hales et al. 2002; Hopp and Foley 2001; Martens et al. 1997; Patz et al. 1998). Such models are useful tools given the lack of long-term mosquito population records (Morin and Comrie 2010). Dynamic models, in particular, can address data constraints by simulating mosquito populations based on local climate and environmental inputs (Focks et al. 1993; Morin and Comrie 2010). This is an important advance in understanding the links between climate change and disease because current and future climate scenarios can be used to simulate local mosquito populations for comparison. At the same time, the complexity of mosquito-borne disease ecology means that not all locations will see an increase in disease, and some may actually experience a decrease under future climate conditions (Lafferty 2009).

Understanding how the geography of dengue outbreaks may change under future climate conditions is a particular concern in the southern United States because it exists along the periphery of transmission in the Americas. Dengue is epidemic and endemic through parts of Central and South America. Although the United States has a history of dengue epidemics through the 1940s, decreased transmission is often attributed to changes in social and built environments (Reiter 2001). However, it remains possible that a more favorable climate may render current strategies less useful. For example, recent dengue outbreaks in Florida, Texas, and Hawaii (Bouri et al. 2012) could be indicative of how a changing climate may enhance transmission risk.

This study addressed these concerns by using downscaled projected climate conditions from global climate model (GCM) ensembles to drive a Dynamic Mosquito Simulation Model (DyMSiM) coupled with a virus transmission component. Model parameter values were derived from successful simulations in San Juan, Puerto Rico (Morin et al. 2015), a nearby location with significant dengue burden and the requisite data necessary for model validation. Present and future meteorological data were produced by a statistical weather generator and were used to drive DyMSiM and to simulate total mosquito populations and human dengue cases for 23 sites in the southeastern United States. These sites were selected because *a*) they are population centers, *b*) they possess relatively complete climatic data sets (> 98%), and *c*) they are regionally at greater risk of tropical disease emergence (Gubler and Clark 1995; Hotez et al. 2014). To isolate the climatic component, the present and future meteorological inputs were the only variables changed among the sites. We simulated mosquito populations and dengue transmission under current and future climate change projections in the southeastern United States to address two primary study questions.

What sites in the southeastern United States can currently support dengue fever transmission, and how might this change under future climate projections?
What impact does climate have on the seasonality of the mosquito vector and on the potential transmission of DENV in the southeastern United States?

## Methods

### Collection and Generation of Climate Data and GCM scenarios

We downloaded 20 years (1981–2000) of observed daily weather station measurements (temperature and precipitation data) from the Global Historical Climatology Network Database (Menne et al. 2012a, 2012b; National Centers for Environmental Information; https://www.ncdc.noaa.gov/oa/climate/ghcn-daily/) for each of the 23 sites. Future climate change data were accessed from GCM data housed in the statistical weather generator LARS-WG5 (LARS) (Rothamsted Research 2016). Daily-level data from GCMs are not directly comparable to observed daily data; therefore, we used LARS to produce a comparable series. LARS uses historical data to create a new synthetic time series that is statistically similar to the observed data at each site, enabling translation between monthly and daily time scales. We evaluated the observed and synthetic time series to assess the software’s ability to reproduce realistic daily data. The daily probability distributions (Kolmogorov–Smirnov test) and monthly means (Student’s *t*-test) for precipitation, minimum, and maximum temperatures for each site showed only one statistically significant value (*p* < 0.01; Jackson, MS, September minimum temperature). The minimal significance differences between the observed and synthetic data sets for these values suggests that the synthetic time series reasonably capture the overall patterns of the observed data sets.

We produced time series for the future climate scenario by calculating GCM ensemble–projected average monthly changes in temperature (absolute) and precipitation (proportion) between the GCM-simulated baseline (1961–1990) and future (2045–2065) periods. We used the closest location for which the GCMs (1.3–3.9° resolution) were run to apply the changes in LARS for each site to the synthetic time series in order to create 21 years of downscaled, daily time series of site-specific future midcentury climate conditions (2046–2056). We applied the same procedure to generate comparable baseline data to use in place of the observed data. We used an ensemble of 15 GCMs (Table 1) to account for models that over- or under-predicted specific variables. The projections used the Intergovernmental Panel on Climate Change (IPCC) SRA1B Special Report on Emissions scenario, which assumes “[a] future world of very rapid economic growth, global population that peaks in mid-century and declines thereafter, and rapid introduction of new and more efficient technologies, with the development balanced across energy sources” (IPCC 2007a). This scenario was selected because it was available for all models and is a midrange emissions and warming scenario (IPCC 2007b).

**Table 1:** The 15 global climate models used to create a single ensemble scenario for each site, obtained through the LARS-WG5 interface (Rothamsted Research 2016).

GCM name	Description
BCM2	Bergen Climate Model (BCM) Version 2
CGMR	Canadian Centre for Climate Modeling and Analysis, CGCM22.1(T47)
CNCM3	Centre National de Recherches Meteorologiques
CSMK3	CSIRO Mark 3.0
FGOALS	LASG, Institute of Atmospheric Physics, Chinese Academy of Sciences
GFCM21	Geophysical Fluid Dynamics Lab, NOAA
GIAOM	NASA Goddard Institute AOM
HADCM3	Hadley Centre for Climate Prediction and Research
HADGEM	Hadley Centre Global Environmental Model
INCM3	Institute of Numerical Mathematics (Russian Academy of Science)
IPCM4	Institut Pierre Simon Laplace (ISPL)
MIHR	National Institute for Environmental Studies, Japan, MRI-CGCM2.3.2
MPEH5	Max-Planck Institute
NCCCSM	NCAR Community Climate System Model
NCPCM	NCAR/NSF/DOE/NASA/NOAA Parallel Climate Model
Notes: AOM, Atmospheric Ocean Model; CSIRO, Commonwealth Scientific and Industrial Research Organisation; DOE, U.S. Department of Energy; GCM, global climate model; LASG, National Key Laboratory of Numerical Modeling for Atmospheric Sciences and Geophysical Fluid Dynamics; NASA, National Aeronautics and Space Administration; NCAR, National Center for Atmospheric Research; NOAA, National Oceanic and Atmospheric Administration; NSF, National Science Foundation.	

### Dynamic Mosquito Simulation Process

DyMSiM is a meteorologically driven, process-based model containing entomological and epidemiological components. The model was first parameterized to simulate *Culex quinquefasciatus* populations (Morin and Comrie 2010) and has now been adjusted to simulate *Ae. aegypti* and DENV development. Using daily temperature and precipitation data, mosquito populations are simulated by calculating daily rates of development and mortality for mosquito cohorts as they proceed through their life cycle (egg, larva, pupa, and adult). Development rates are calculated using temperature, and mortality rates are dependent on temperature and, in the case of larvae and pupae, water availability. Water habitat is calculated for rain-filled containers (precipitation minus evaporation and spilling) and permanent water sources (levels remain constant). During the adult stage, cohorts of mosquitoes proceed through their gonotrophic cycle, including blood meal questing, ovarian development, and egg laying. Rates of feeding and ovarian development are temperature dependent. Once ovarian development is complete and water is available, the eggs are deposited. During feeding, mosquitoes can become infected with DENV at a probability based on human infection rates (see below) and will then proceed through a temperature-regulated EIP. We input new cohorts of eggs into the model if the mosquito population drops to zero to prevent extinction of the mosquito within the model during inhospitable climate conditions.

The human population is simulated using a compartmental susceptible–exposed–infectious–recovered model. Susceptible humans move to the exposed stage based on the number of infected mosquitoes that have completed the EIP. Exposed individuals remain in the exposed and infectious stages for a period of 5–7 days before recovering from the infection. Mosquito infection rates are calculated using the number of humans in the infectious stage. To prevent extinction of the virus, a minimum human infection rate is used when there are few or no infections in the human population. The governing equations for each stage (mosquito and human) as well as the parameters and their equations can be found in Morin et al. (2015).

San Juan was used for model evaluation because of the availability of multiyear dengue case records, which are unavailable in the mainland United States owing to a lack of long-term transmission. The climate of San Juan is an important driver of local transmission, and DyMSiM can effectively capture inter- and intra-annual variability (Morin et al. 2015). We used the same predictors (7 variables, 96 total parameter values, Table 2) that had been applied in the previous analysis of transmission in San Juan for the present analysis, and although we acknowledge the potential for variation among individual locations, it was not possible to validate these parameters for each location in the present analysis. In addition, standardizing model outputs for each U.S. site against San Juan data while holding all model parameters other than climate constant allowed us to estimate the specific effects of climate and climate change on dengue transmission.

**Table 2:** Parameter names, values, and total number of values used to create the 96 different model parameterizations (Morin et al. 2015).

Parameter name (units)	Values	No. of values
Habitat area (cm^2^)^*a*^	1.0 × 10^7^, 1.4 × 10^7^, 1.8 × 10^7^, 2 × 10^7^, 2.4 × 10^7^, 2.6 × 10^7^, 2.8 × 10^7^, 5.0 × 10^7^, 6.0 × 10^7^, 7.0 × 10^7^, 9.0 × 10^7^, 1.0 × 10^8^	12
Container height (cm)^*b*^	8, 12	2
Minimum human infection rate (fraction of infectious humans in the population)	4 × 10^–5^, 6 × 10^–5^, 8 × 10^–5^	3
Maximum larval density (per cm^3^)	0.5, 1	2
Adult survival rate (fraction of mosquitoes surviving per day)	0.86, 0.88	2
Length of human infectious period (days)	5, 7	2
Maximum mammal transmission probability^*c*^	0.5, 1	2
^***a***^Habitat area refers to the surface area of water containers (filled either manually or by precipitation). ^***b***^Container height refers to the maximum height of water in a container before additional precipitation will cause spillover. ^***c***^Maximum mammal transmission probability refers to the highest probability of transmission of the virus from mosquito to human during a blood meal dependent on temperature.		

The daily meteorological data sets generated by LARS, described above, were used to drive DyMSiM to simulate current and projected future mosquito populations and human dengue cases. DyMSiM also requires the parameterization of additional environmental variables and the site latitude to determine the duration of sunlight hours (which influences the evaporation of standing water). To account for spatiotemporal variability in parameter values and climate conditions, 96 simulations were performed at each site using different combinations of model parameter values. The parameter values were selected to represent ranges of values reported in the literature (Morin et al. 2015). Six of the seven parameters included in our suite of simulations were represented by only 2 or 3 possible values because their values are well established (Table 2). In contrast, we included 12 possible values for habitat area (determined by the number and surface area of containers available for egg laying) because of greater uncertainty and potential for variation in this parameter (Morin et al. 2013, 2015). Parameter values for the simulation were chosen from the best-fit 1% of simulations for San Juan, Puerto Rico for the period 2010–2013 because of their superior ability to replicate patterns of dengue (Morin et al. 2015) (Table 2). We simulated a total of 21 years of daily total mosquito populations and dengue case data for each site driven by baseline climate, and we repeated this process with the future climate scenarios. After discarding the first year of DyMSiM results to allow for model spin-up time, each of the 23 mainland U.S. sites had four associated 20-year daily time series: *a*) total mosquitoes, present climate; *b*) total dengue cases, present climate; *c*) total mosquitoes, future climate; and *d*) total dengue cases, future climate.

### Analysis and Map Visualization Process

We compared the outputs of the model runs with output from San Juan for each site. The analysis results are indicative of one of two possibilities for each location: *a*) transmission in the mainland U.S. site occurs at the same level as that in San Juan, indicating that the climate is suitable for transmission, but socioeconomic factors, lack of importation, or other unaccounted-for factors are limiting transmission; or *b*) dengue transmission in the mainland U.S. site is significantly reduced or absent compared with that in San Juan, suggesting that the site climate is not suitable for epidemic dengue. By repeating these steps for the future climate scenarios, we also assessed future transmission potential for each location.

**Figure 1:**
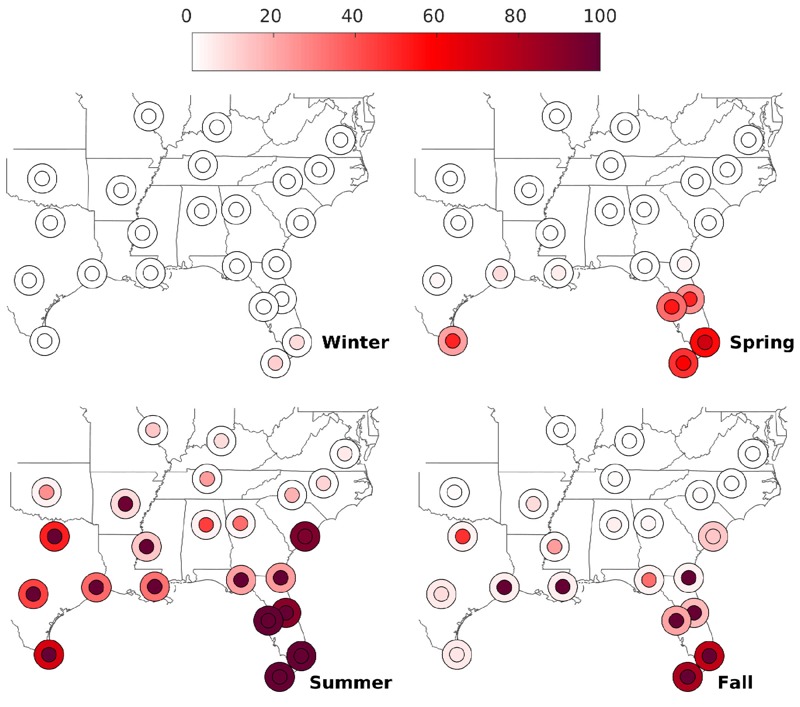
Baseline and future dengue cases by season (Winter: January–March; Spring: April–June; Summer: July–September; Fall: October–December). The larger circles denote present dengue, and the inner circles denote projected future dengue. The scale bar refers to percent of dengue cases compared with San Juan, Puerto Rico model output for the same period. The sites are Atlanta, Georgia; Birmingham, Alabama; Brownsville, Texas; Charleston, South Carolina; Charlotte, North Carolina; Dallas, Texas; Jackson, Mississippi; Jacksonville, Florida; Key West, Florida; Little Rock, Arkansas; Louisville, Kentucky; Miami, Florida; Nashville, Tennessee; New Orleans, Louisiana; Oklahoma City, Oklahoma; Orlando, Florida; Port Arthur, Texas; Raleigh, North Carolina; Richmond, Virginia; San Antonio, Texas; St. Louis, Missouri; Tallahassee, Florida; and Tampa, Florida.

**Figure 2:**
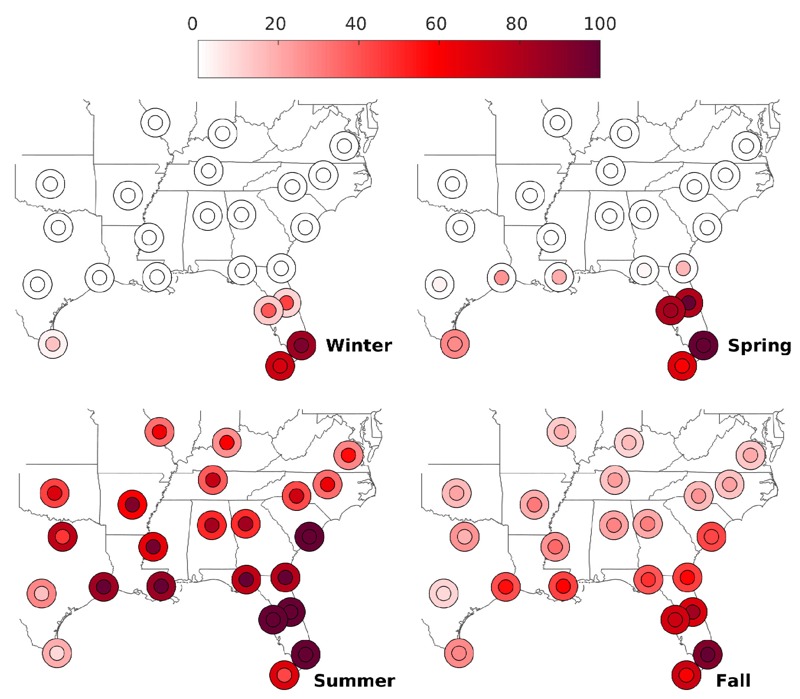
Baseline and future mosquito populations by season (Winter: January–March; Spring: April–June; Summer: July–September; Fall: October–December). The larger circle denotes present mosquito populations, and the inner circle denotes projected future mosquito populations. The scale bar refers to percent of mosquitoes compared with San Juan, Puerto Rico model output for the same period.

Maps were created from the model output to visualize spatio-temporal variance. For visualization purposes, the daily model outputs (mosquito population and dengue cases) were aggregated to seasonal values by dividing the year into four 13-week periods (approximately January–March, April–June, July–September, and October–December). For each mainland U.S. location and parameterization, data values were averaged across the 20-year runs and were then compared with the corresponding data for San Juan, Puerto Rico. The comparison metric (percent of value in San Juan) was then averaged across the parameterizations and mapped (Figures 1 and 2).

In addition to estimating mosquito populations and dengue cases during each season for the 23 locations, we conducted a separate analysis to estimate weekly variation in dengue cases for the Key West, Florida and Brownsville, Texas sites. Specifically, we averaged estimated numbers of weekly dengue cases across the 20-year period to create a single annual time series (at weekly resolution) for each simulation and location. The time series were then standardized against the cumulative annual San Juan dengue cases using the following equation:


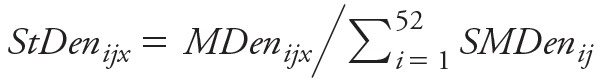


*StDen =* standardized dengue cases, *MDen* = modeled dengue averaged over the 20 years, *SMDen =* modeled dengue in San Juan averaged over the 20 years, *i* = epidemiological week, *j* = simulation number, *x* = site. An epidemiological week (epi week) is a standardized way of defining the aggregation of days into weeks so that data are comparable across years. We then averaged across all the simulations to create one annual time series each for Key West and Brownsville under the base and future climate scenarios. This method is similar to the method used to estimate seasonal averages for all 23 locations. Here, however, the standardization process only serves to highlight the seasonality of transmission (i.e., the percent of the total annual dengue cases that occurred each week).

## Results

Our estimates suggest that under baseline climate conditions, dengue transmission may be possible in several sites in the southeast United States (Figure 1). The estimated transmission potential was highest during summer (July–September), although some locations in Florida and Texas may have transmission during the spring and fall as well. In addition, our estimates suggest that southern Florida is as climatically capable of supporting dengue transmission as Puerto Rico during the summer months, denoted by estimated values that are 100% of values estimated for San Juan. Absent caseloads during the winter at all 23 sites suggest that for all locations, the current winter temperatures are too low to allow virus transmission. However, our estimates suggest that mosquito populations during the winter in southern Florida may be only slightly lower than mosquito populations in San Juan (Figure 2). Although our model estimates suggest that *Ae. aegypti* populations may be present at the northernmost sites during the summer and the fall, there is little likelihood of local transmission (dengue cases) during any season in these locations.

Our simulations suggest similar seasonal and regional patterns under projected future scenarios. Estimated numbers of cases (transmission potential) are highest during the summer, and a larger number of northern sites show some potential for transmission during the summer (Figure 1). In the far South, the numbers of estimated cases are similar to those in San Juan during the summer, and some transmission could continue in the fall and the spring. Model projections also indicate an increase in the length of the adult mosquito season, but with less of an increase in the number of sites affected compared with the estimated expansion of transmission potential (Figure 2).

### Site-Specific Results: Key West, Florida, and Brownsville, Texas

**Figure 3:**
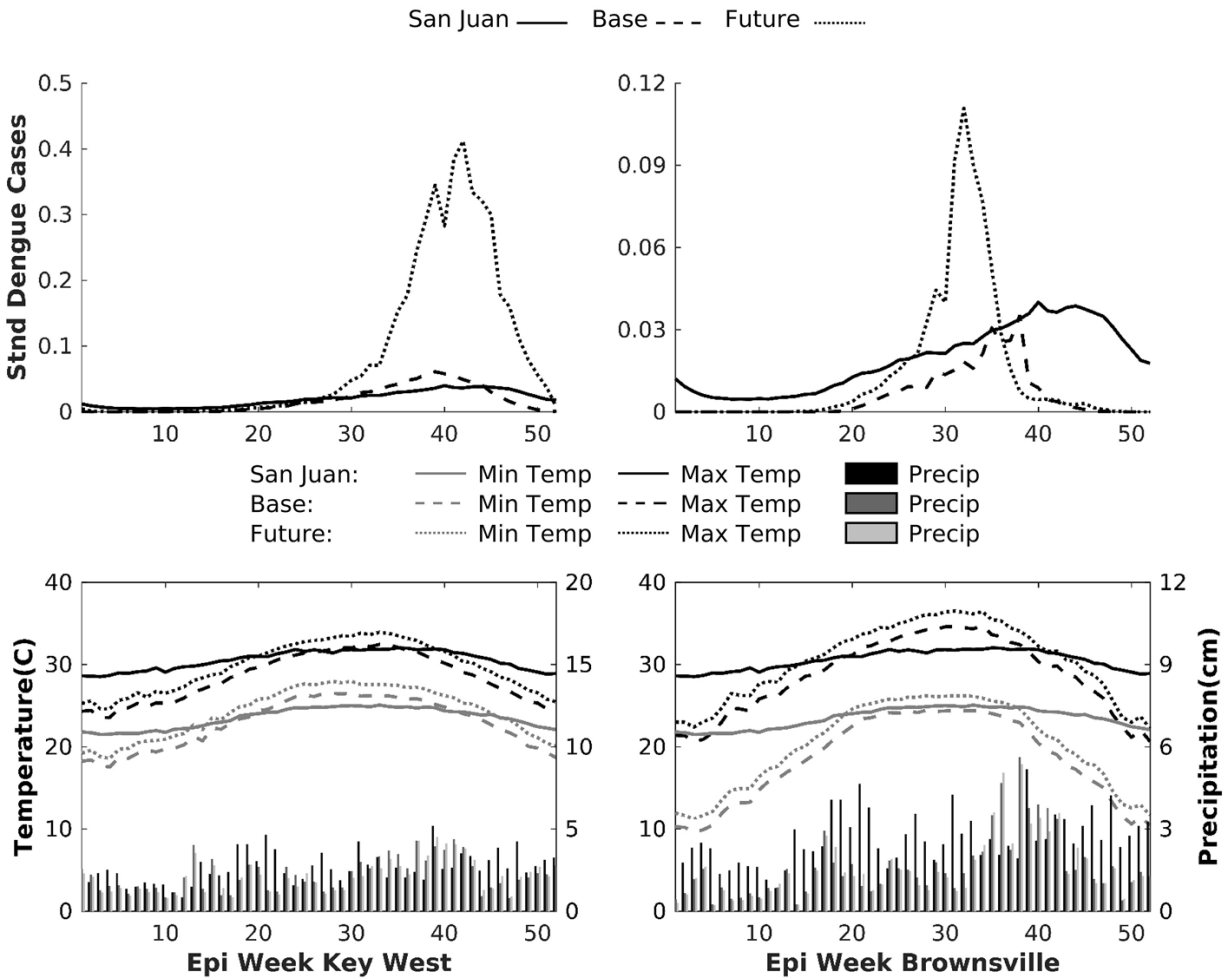
The top panel shows the weekly (averaged across simulations and years) dengue cases (percent of Puerto Rico annual total dengue) for San Juan, Puerto Rico and the baseline and future scenarios for Key West, Florida and Brownsville, Texas. The bottom panel shows the corresponding temperature and precipitation values. Epi week, epidemiological week.

We conducted additional analysis on Key West, Florida, and Brownsville, Texas, because both have had recent dengue cases (see Bouri et al. 2012) but have contrasting climate characteristics. Figure 3 shows dengue cases at these sites as a percentage of total cases in Puerto Rico, along with plotted climate data. Presently, Key West climate conditions are more favorable for transmission than those in San Juan during the summer (weeks 30–44), with the window widening to weeks 27–51 under the future scenario. Our modeled output data showed, on average, low but continual transmission throughout the winter in San Juan. However, this year-round transmission is not maintained in Key West or Brownsville (Figure 1). The climate data in Figure 3 show that San Juan has a smaller annual temperature range than these two sites, with warmer winter temperatures capable of supporting year-round transmission. Conversely, cooler winter conditions are likely to prevent or limit transmission in Key West, even under future climate conditions. Estimates show a similar pattern for Brownsville, but with a shorter time window for transmission that does not currently exceed that of San Juan. However, transmission is projected to increase in future climate scenarios, exceeding current case numbers in San Juan during the summer (weeks 27–35). Nevertheless, temperatures remain substantially cooler than in San Juan during fall and winter, thus limiting the potential for year-round transmission.

## Discussion

Our results suggest that the current climate is capable of supporting dengue transmission throughout much of the southeastern United States during limited periods of the summer months. Evidence of climatic suitability for dengue is not surprising given historic epidemics in this region. According to our model, southern Florida has the highest likelihood of transmission, consistent with the fact that Florida has reported locally-acquired cases every year since 2009 [Khan 2016; U.S. Geological Survey (USGS) 2016]. Our estimates for Brownsville under baseline climate conditions also indicate a relatively long infectious mosquito season, consistent with current data for dengue transmission in this location (Bouri et al. 2012).

Although our baseline climate projections suggest that the potential for dengue transmission in southern Florida during the summer is similar to that in San Juan, outbreaks are much less common in southern Florida than in San Juan. This difference is likely due in part to differences in social factors that influence transmission, such as housing infrastructure and public health services, which may also mitigate future risk. However, our estimates suggest that environmental factors may also play a role: specifically, the climate in south Florida appears to be too cold to maintain regular dengue transmission throughout the winter months, whereas transmission is sustained year-round in Puerto Rico, albeit at lower levels during the winter.

Our model projections for future climate scenarios suggest that the potential for dengue transmission will continue to be seasonal throughout the southeastern United States, without becoming a year-round phenomenon except perhaps in southern Florida, which may have some winter dengue activity. This scenario could increase the possibility of virus maintenance throughout the winter, at least during warmer-than-average years. Our estimates also suggest that the length of the potential transmission season will increase for most sites. Although our projections suggest a small possibility of summer dengue transmission at northern sites that currently do not support it (e.g., Virginia, North Carolina, Tennessee, Kentucky, and Missouri), time windows for transmission would be short, and at most, only a few localized cases would be expected to occur.

In all locations considered, and for both current and future scenarios, the projected time window for dengue transmission is shorter than the seasonal time window for *Ae. aegypti* populations. Studies show that the EIP shortens as temperatures rise > 26°C (Watts et al. 1987; Rohani et al. 2009). The ideal temperature range for *Ae. aegypti* is as low as 20°C (Tun-Lin et al. 2000), but the mosquito can remain active until temperatures drop to between 10°C–15°C (Christophers 1960). Our results suggest that conditions favorable to the virus (when simulated dengue cases occur) only arise during the warmest times of the year, likely because of the lengthening of the EIP at lower temperatures. Furthermore, our model suggests that even in locations where mosquitos may survive year-round, such as southern Florida, temperatures are too low to permit dengue transmission during an infected mosquito’s lifespan during much of the year. Therefore, outbreaks occur only when dengue is reintroduced during favorable conditions, that is to say, when the length of the EIP is shorter than the lifespan of the mosquito. In most U.S. locations, dengue outbreaks are rare because this potential time window is very short, whereas cases are more common in southern Texas and Florida, where the time window for conditions that favor transmission is longer.

Although our findings suggest that current climate conditions during the summer in southern Florida are capable of supporting dengue transmission at levels similar to those in San Juan, dengue outbreaks are much less common in southern Florida. Furthermore, although our model projections suggest that lower winter temperatures and a lack of year-round transmission contribute to this difference, social factors, public health infrastructure, and other influences may also play a role and have been proposed to explain the decline in dengue fever transmission in the southern United States over time. Nonetheless, the likely influence of these factors on the risk of dengue does not negate the potential for an increase in transmission under more favorable climate conditions in the future, and our findings suggest that public health departments should be prepared to adapt to new levels of risk that may result from longer mosquito seasons with wider transmission windows.

Geographic proximity also plays a role in disease transmission and spread. Places with significant tourism, such as southern Florida, or places with increased migration between endemic and nonendemic locations, such as the U.S.-Mexico border region, have recently experienced localized dengue outbreaks (Adalja et al. 2012). Therefore, if dengue were to become endemic in Florida, or if it were to at least overwinter during some years, the risks to neighboring states could increase considerably.

### Limitations and Future Work

A number of important factors known to affect dengue, such as humidity, herd immunity, vector competition, insect resistance, viral mutation, and socioeconomic factors, are not included in the model. Nor do we account for land cover variation, geographic relationships to endemic areas, or potential human adaption strategies. Dengue disease dynamics are complex, and future research should aim to develop more comprehensive models that can better assess the roles of such variables.

In light of the abovementioned factors, our results should be interpreted as climate-based projections of relative differences in estimated risks, not as concrete predictions of future climate change impacts on dengue fever. The generated climate data provide only possible values for daily temperature and precipitation. Synthetic baseline and GCM data sets are inherently subject to inaccuracies; for example, some standard deviation metrics between the observed and synthetic data sets did not perform as well as the evaluation metrics described in “Methods.” We attempted to partially address this concern by using the synthetic baseline data in place of the observed data to standardize such inaccuracies across the baseline and future time series for comparative purposes. The use of larger data sets and improved downscaling techniques may improve the accuracy of future studies.

Case studies have shown that the spatial distribution of *Ae. aegypti* is heterogeneous within cities and regions (Hayden et al. 2010; Murray et al. 2013). We have attempted to account for this heterogeneity by using the average from 96 different parameterizations that were selected to optimize model performance based on an analysis of data from Puerto Rico. Nevertheless, it remains possible that some locations in our study may have different spatial patterns of breeding sites than those captured in the San Juan validation, and readers should keep in mind that these results were modeled using San Juan parameters. Further field studies are needed to quantify the distribution of *Ae. aegypti* across U.S. urban landscapes.

Although it would be ideal to have accurate mosquito population and breeding site data for each location, the paucity and accuracy of such records and the length of time needed to generate methodologically sound data sets is an ongoing problem in climate and health research. DyMSiM seeks to fill such a void by generating environmentally driven mosquito populations in the absence of mosquito data. Future work should be focused on evaluating model performance in multiple locations and on obtaining better location-specific information for model parameterization; the model should be periodically re-validated as larger dengue data sets become available.

Finally, more detailed risk assessments are needed to better understand site-specific vulnerabilities. This may include parameterizing the model against local mosquito data, if available, or running the model at finer spatial scales within a city. Given that microclimatic variations within a community can affect mosquito abundance (Hayden et al. 2010), this additional detail may be useful for determining local risk. Inter- and intra-annual future climate variations may also affect the seasonality of dengue transmission in ways that current GCMs cannot account for when downscaled to the local level. As GCM capabilities improve, along with our understanding of shifting weather patterns, additional analysis may be useful for understanding dengue transmission potential.

These limitations are inherent in modeling approaches. Nevertheless, this technique is useful for demonstrating the contributing role of climate in shaping dengue fever transmission risk within the southeastern United States. The impact of climate on *Ae. aegypti* abundance patterns is also important for the Chikungunya virus and the Zika virus. The current DyMSiM model does not incorporate specific characteristics of these viruses, but the potential for an increase in *Ae. aegypti* seasonality may have implications for the transmission of these viruses as well. Future studies incorporating specific temperature thresholds for the Zika virus and the Chikunguyna virus would be helpful.

## Conclusion

Hosking and Campbell-Lendrum (2012) noted a lack of studies quantifying the links between climate and human health; this is particularly true for analyses of future dengue fever in the southeastern United States. We used a dynamic modeling approach to estimate future impacts of climate change on *Ae. aegypti* and dengue cases in the United States. Our results highlight the potential influence of climate on both the vector and the virus. Some locations may see an increase in both disease risk and vector abundance, whereas others may see an increase in *Ae. aegypti* populations but remain on the fringe of dengue transmission. Our estimates suggest that the dengue transmission window is narrower than the *Ae. aegypti* season length at all of the locations evaluated, consistent with stringent climatic limitations on the virus. Although social and public health infrastructure play an important role in preventing transmission, this research shows that current climatic conditions may also be limiting the virus. Our findings indicate that it is too cold during the winter months for viral transmission to be sustained under present mainland U.S. climate conditions. If so, virus reintroduction is required for dengue outbreaks to occur. However, future climate changes may expand the transmission potential in the southeastern United States, making dengue a public health challenge in the future.
